# Supplementary figures and images for: MetGEMs Toolbox: Metagenome-scale models as integrative toolbox for uncovering metabolic functions and routes of human gut microbiome
Source: PLoS Comput Biol. 2021 Jan 6;17(1):e1008487. doi: 10.1371/journal.pcbi.1008487 (PMC7787440; doi:10.1371/journal.pcbi.1008487)

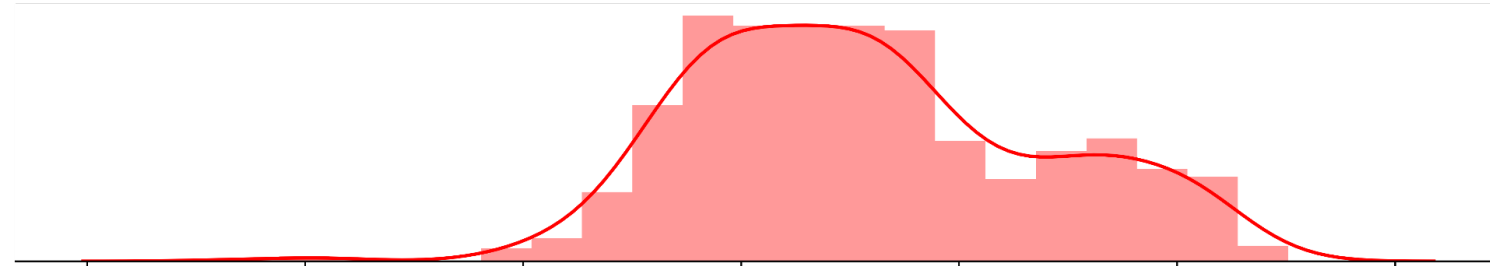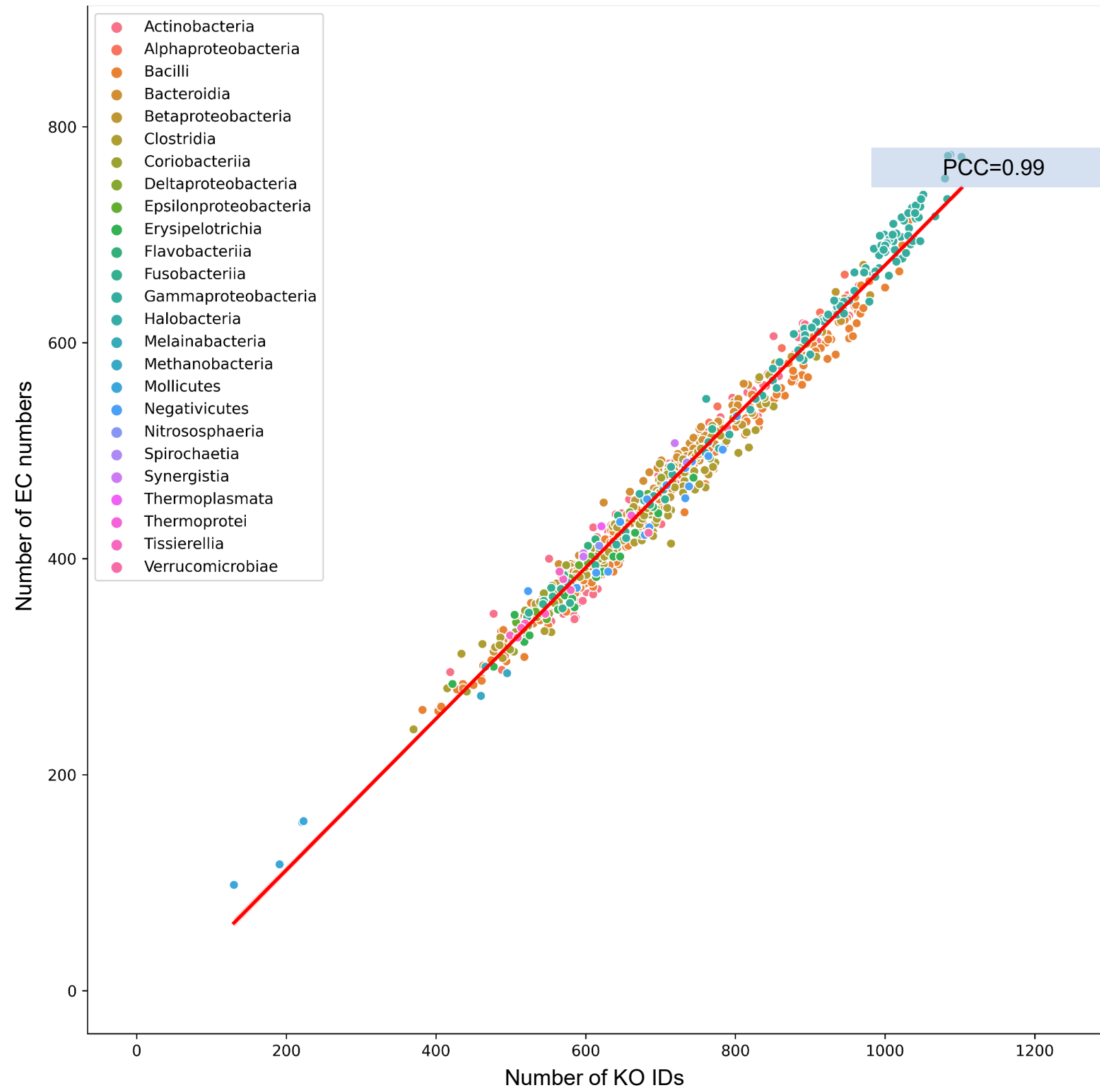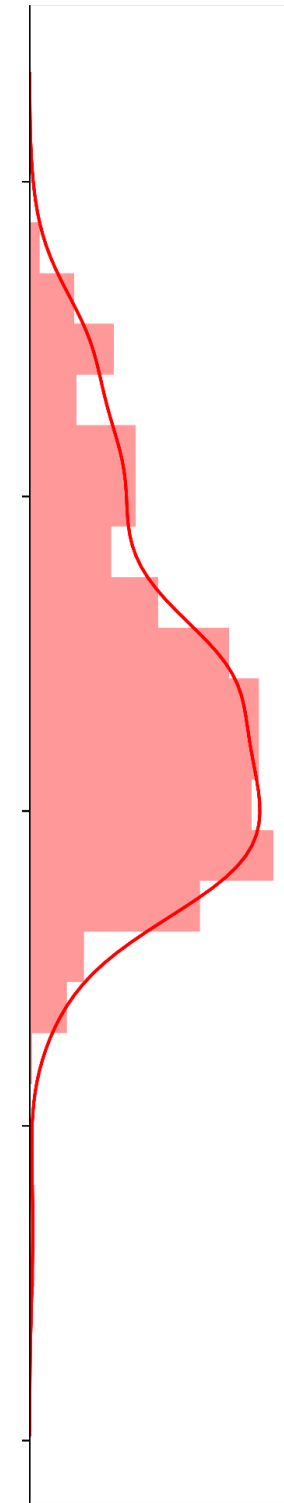

Supplement: S1 Fig — (PDF) [file pcbi.1008487.s001.pdf]

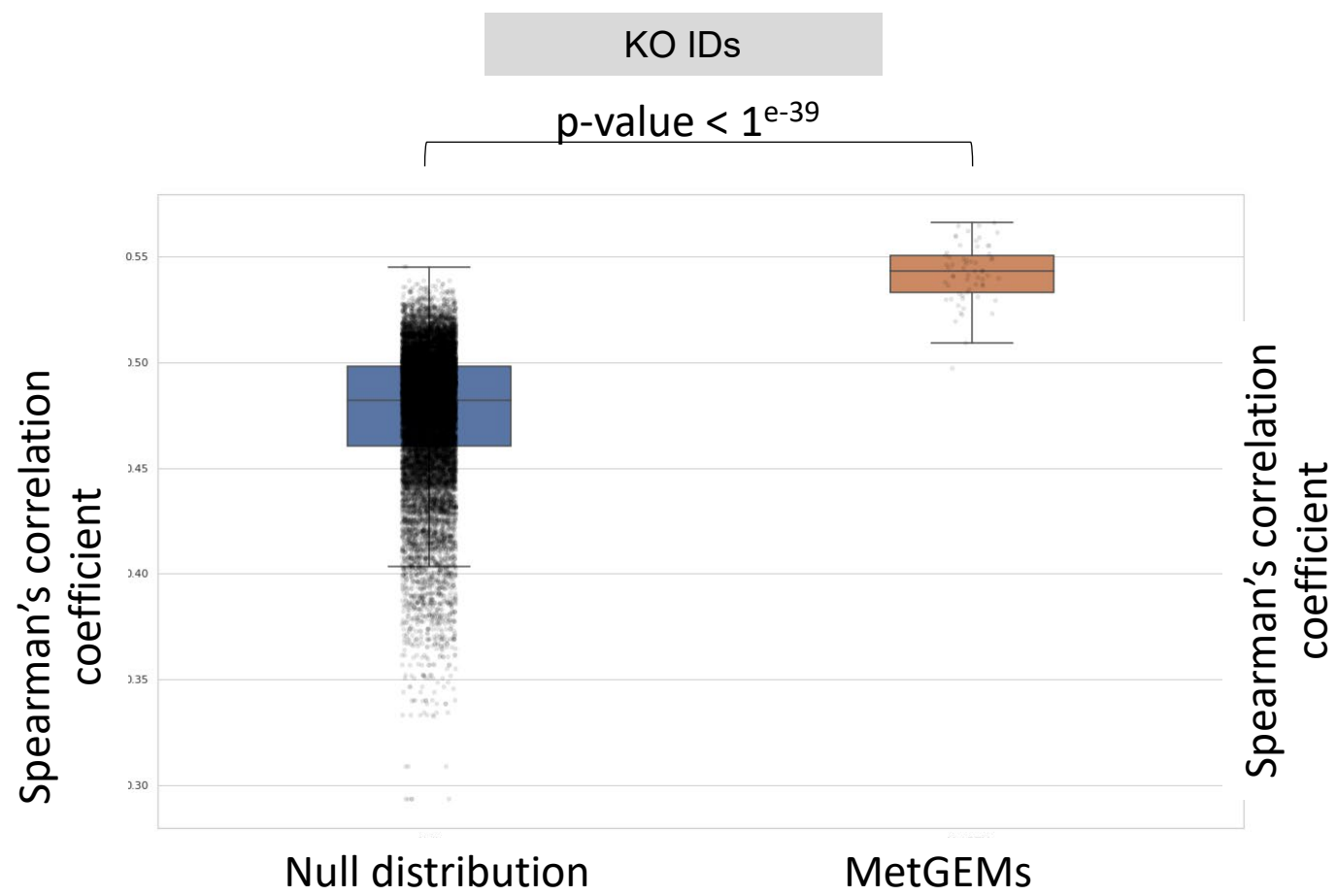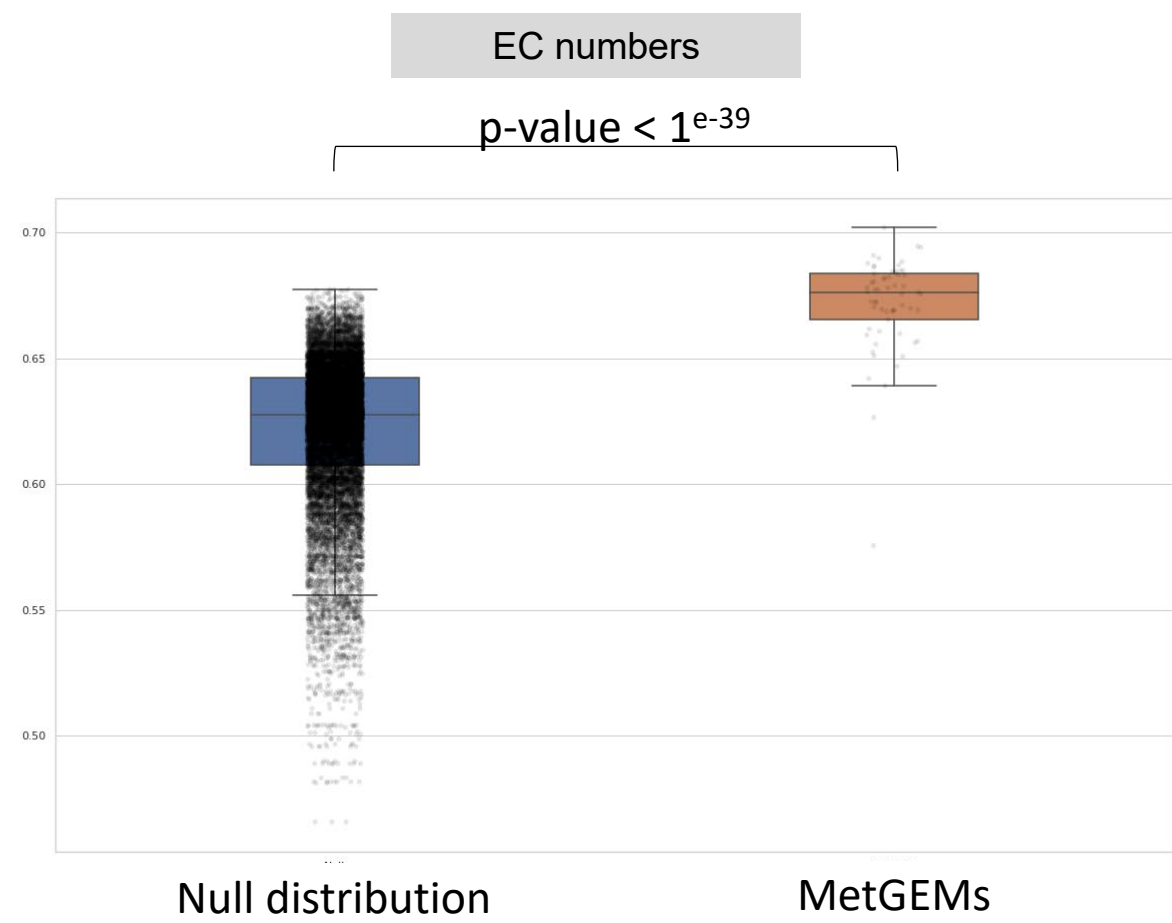

Supplement: S2 Fig — Comparison are shown between results from real dataset and null distribution from permutation and bootstrap sampling. Independent T-test was used to assess the differences of (A) KO IDs prediction (B) EC numbers prediction. (PDF) [file pcbi.1008487.s002.pdf]

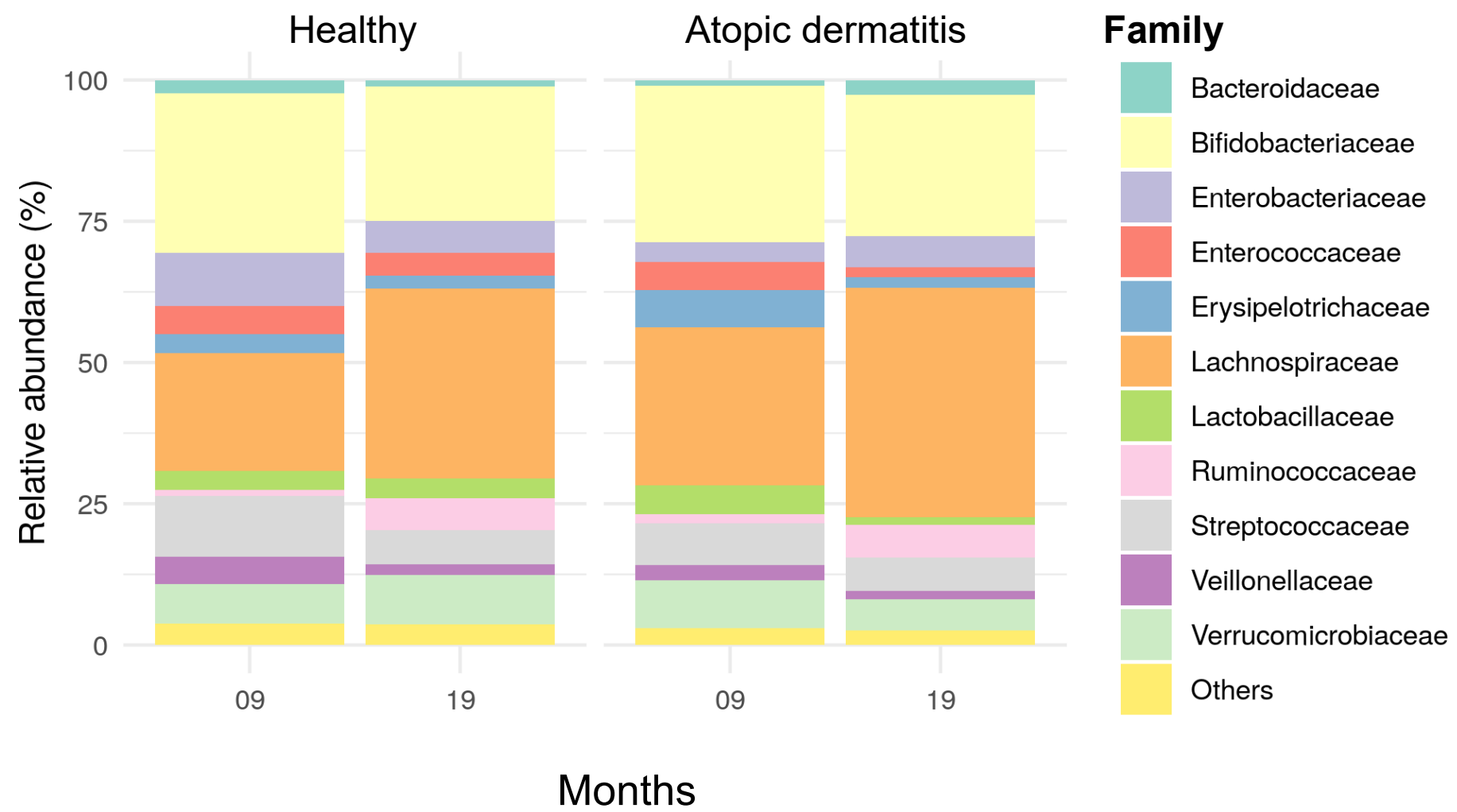

Supplement: S3 Fig — (PDF) [file pcbi.1008487.s003.pdf]
