# Supplementary material for: MetGEMs Toolbox: Metagenome-scale models as integrative toolbox for uncovering metabolic functions and routes of human gut microbiome
Source: PLoS Comput Biol. 2021 Jan 6;17(1):e1008487. doi: 10.1371/journal.pcbi.1008487 (PMC7787440; doi:10.1371/journal.pcbi.1008487)
Supplement: S4 Fig — (PDF) [file pcbi.1008487.s004.pdf]

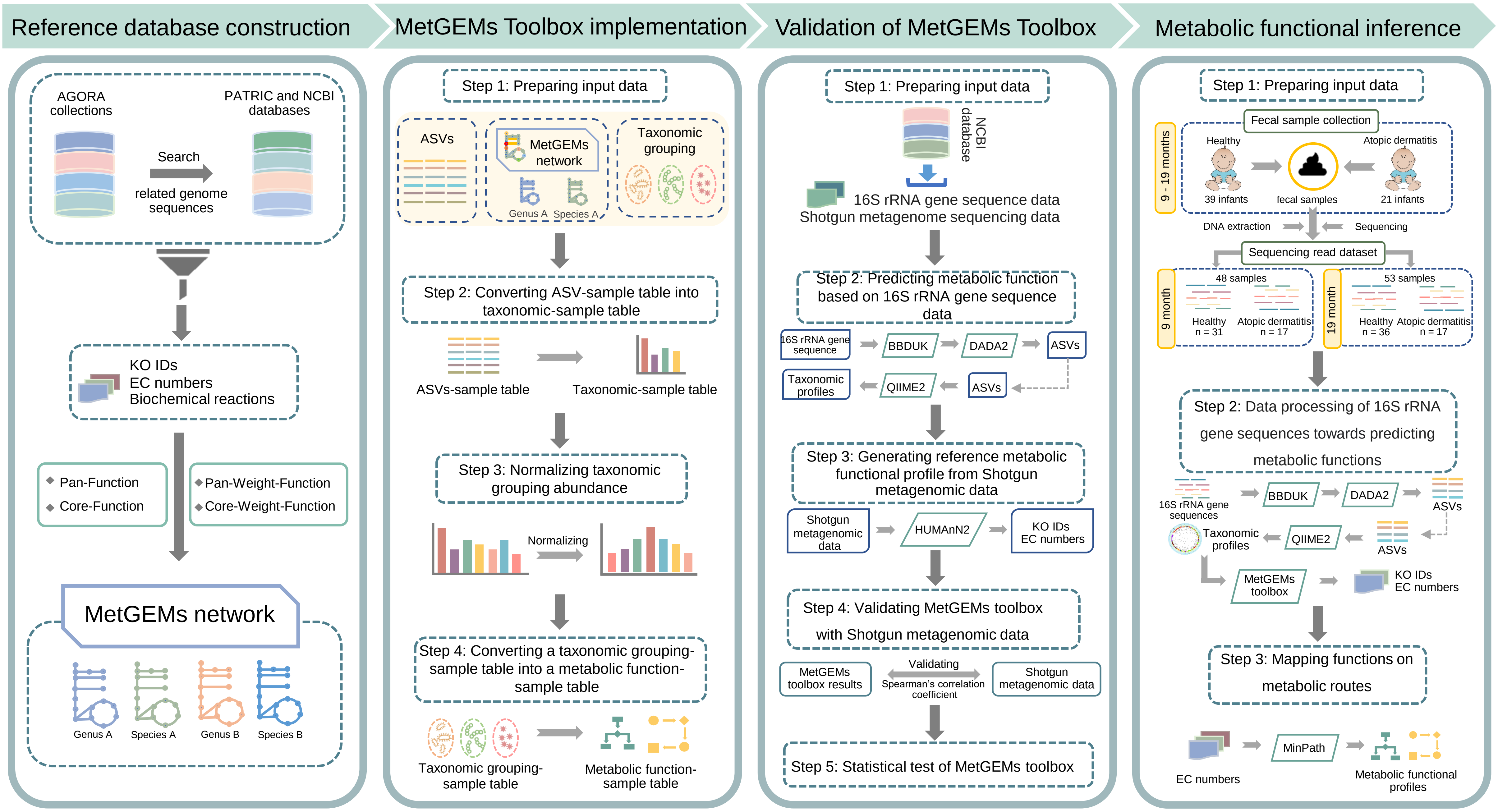

Reference database construction
MetGEMs Toolbox implementation
Validation of MetGEMs Toolbox
Metabolic functional inference
AGORA collections
PATRIC and NCBI databases
Search
related genome sequences
KO IDs
EC numbers
Biochemical reactions
Pan-Function
Core-Function
Pan-Weight-Function
Core-Weight-Function
MetGEMs network
Genus A
Species A
Genus B
Species B
Step 1: Preparing input data
ASVs
MetGEMs network
Taxonomic grouping
Step 2: Converting ASV-sample table into taxonomic-sample table
ASVs-sample table
Taxonomic-sample table
Step 3: Normalizing taxonomic grouping abundance
Normalizing
Step 4: Converting a taxonomic grouping-sample table into a metabolic function-sample table
Taxonomic grouping-sample table
Metabolic function-sample table
Step 1: Preparing input data
NCBI database
16S rRNA gene sequence data
Shotgun metagenome sequencing data
Step 2: Predicting metabolic function based on 16S rRNA gene sequence data
16S rRNA gene sequence
BBDUK
DADA2
ASVs
Taxonomic profiles
QIIME2
ASVs
Step 3: Generating reference metabolic functional profile from Shotgun metagenomic data
Shotgun metagenomic data
HUMAnN2
KO IDs
EC numbers
Step 4: Validating MetGEMs toolbox with Shotgun metagenomic data
MetGEMs toolbox results
Validating
Spearman's correlation coefficient
Shotgun metagenomic data
Step 5: Statistical test of MetGEMs toolbox
Step 1: Preparing input data
Fecal sample collection
9 - 19 months
Healthy
Atopic dermatitis
39 infants
fecal samples
21 infants
DNA extraction
Sequencing
Sequencing read dataset
9 month
48 samples
Healthy n = 31
Atopic dermatitis n = 17
19 month
53 samples
Healthy n = 36
Atopic dermatitis n = 17
Step 2: Data processing of 16S rRNA gene sequences towards predicting metabolic functions
16S rRNA gene sequences
BBDUK
DADA2
ASVs
Taxonomic profiles
QIIME2
ASVs
MetGEMs toolbox
KO IDs
EC numbers
Step 3: Mapping functions on metabolic routes
EC numbers
MinPath
Metabolic functional profiles
