# Supplementary material for: MetGEMs Toolbox: Metagenome-scale models as integrative toolbox for uncovering metabolic functions and routes of human gut microbiome
Source: PLoS Comput Biol. 2021 Jan 6;17(1):e1008487. doi: 10.1371/journal.pcbi.1008487 (PMC7787440; doi:10.1371/journal.pcbi.1008487)
Supplement: S1 Doc — (PDF) [file pcbi.1008487.s009.pdf]

S1 Doc. Supplementary Methods of MetGEMs toolbox development

This document describes all details on how we process each NGS dataset in the main text. While main text already explained tools and options used briefly, this document describes all details we used in our analysis in this document to encourage the practice of reproducibility in research. We understand that having the document like this not the perfect solution for reproducibility problem, but we need to avoid the issue of data ownerships, licenses, and some specific technical difficulties, hence, we decided on the document.

All the analyses were done on CentOS Linux 6.7. All tools used here were installed via Bioconda with python 3.8 as the base package. The options and arguments used in each tool are all default unless noted. All data of same type (e.g. amplicon, shotgun) were filtered and processed under the same pipeline and tool to ensure the comparability between datasets.

Preprocessing

To reduce the technical differences between NGS datasets, all datasets were filtered and trimmed using the BBDUK (v38.12) [1] with the options (*ktrim=l k=13 mink=6 hdist=1 restrictleft=25 copyundefined=T literal=PRIMERSEQUENCES*). The primer sequences which we retrieved from the literature. In case of paired read, we only keep the read that both forward and reverse pass the filtered.

Amplicon (single) 454 reads processing

This was done to the HMP1 gut microbiome dataset (16s rRNA gene sequencing) [2] as described in the main text.

The reads that pass filtered were denoised using script included within qiime-dada2's repository (https://github.com/qiime2/q2-dada2/blob/bd2b0607b57a02824dd00c23024aa9631e2c9a49/q2_dada2/assets/run_dada_single.R). We used default option for trim (trunc_len=0, trim_left=0), filter (max_ee=2.0), and denoise (n_reads_learn =250000). The denoised reads were then assigned with QIIME2 (v2019.1) [3] command *feature-classifier classify-sklearn* with Greengenes database (v13.8) [4].

The results were used for functional analysis with PICRUSt2 (v2.1.0-b) [5] and MetGEMs (commit: 42d402c41225d4f5638d6615e8b6144f9a3d6efb). For PICRUSt2, *picrust2_pipeline.py* script was used. The MetGEMs was run using *markp* commands with all included models.

Amplicon (paired) illumina reads processing

The paired reads that pass filtered were denoised using script included within qiime-dada2's repository (https://github.com/qiime2/q2-dada2/blob/bd2b0607b57a02824dd00c23024aa9631e2c9a49/q2_dada2/assets/run_dada_paired.R). The option for trim (trunc_len=0, trim_left=0), filter (max_ee=2.0), denoise (n_reads_learn=250000), and mergePairs (defaults) were used. The merged reads were then

assigned with QIIME2 (v2019.1) [3] command *feature-classifier classify-sklearn* with Greengenes database (v13.8) [4].

The results were used for functional analysis with PICRUSt2 (v2.1.0-b) [5] and MetGEMs (commit: 42d402c41225d4f5638d6615e8b6144f9a3d6efb). For PICRUSt2, *picrust2_pipeline.py* script was used. The MetGEMs was run using *markp* commands with all included models.

Shotgun metagenomics sequencing data processing

We checked for the host contaminate using Bowtie2 [6] but we found less than 100 reads for each sample and decided to not filter them out. The functional analysis was done using HUMAnN2 (version 0.11.2) [7] with chocophlan's database (v201901) and uniref90_annotated (v2001901).

The forward, reverse and unpaired reads were concatenated into single file. Then, HUMAnN2 [7] was used run to identify the abundances of annotated UniRef90 gene families using *–search-mode uniref90*. The abundances of UniRef90 gene families were regrouped into KO IDs and EC numbers using *humann2_regroup_table*.

References

1. Bushnell B. 2014. BBTools software package. https://sourceforge.net/projects/bbmap/

2. Huttenhower, C., Gevers, D., Knight, R., Abubucker, S., Badger, J. H., Chinwalla, A. T., ... & Giglio, M. G. (2012). Structure, function and diversity of the healthy human microbiome. *Nature*, 486(7402), 207.

3. Bolyen, E., Rideout, J. R., Dillon, M. R., Bokulich, N. A., Abnet, C. C., Al-Ghalith, G. A., ... & Bai, Y. (2019). Reproducible, interactive, scalable and extensible microbiome data science using QIIME 2. *Nature biotechnology*, 37(8), 852-857.

4. McDonald, D., Price, M. N., Goodrich, J., Nawrocki, E. P., DeSantis, T. Z., Probst, A., ... & Hugenholtz, P. (2012). An improved Greengenes taxonomy with explicit ranks for ecological and evolutionary analyses of bacteria and archaea. *The ISME journal*, 6(3), 610-618.

5. Douglas, G. M., Maffei, V. J., Zaneveld, J. R., Yurgel, S. N., Brown, J. R., Taylor, C. M., ... & Langille, M. G. (2020). PICRUSt2 for prediction of metagenome functions. *Nature Biotechnology*, 1-5.

6. Langmead, B., & Salzberg, S. L. (2012). Fast gapped-read alignment with Bowtie 2. *Nature methods*, 9(4), 357.

7. Franzosa, E. A., McIver, L. J., Rahnavard, G., Thompson, L. R., Schirmer, M., Weingart, G., ... & Huttenhower, C. (2018). Species-level functional profiling of metagenomes and metatranscriptomes. *Nature methods*, 15(11), 962-968.
