# Supplementary material for: MetGEMs Toolbox: Metagenome-scale models as integrative toolbox for uncovering metabolic functions and routes of human gut microbiome
Source: PLoS Comput Biol. 2021 Jan 6;17(1):e1008487. doi: 10.1371/journal.pcbi.1008487 (PMC7787440; doi:10.1371/journal.pcbi.1008487)
Supplement: Attachment — Submitted filename: Answers_reviewers_comments.docx [file pcbi.1008487.s010.docx]

**Comments to the Authors:
Please note here if the review is uploaded as an attachment.**

Reviewer #1:

This work proposes the MetGEMs as integrative toolbox which can predict the functional capabilities of microbiome from amplicon sequencing data. The proposed method improved over the existing method by applying genome-scale metabolic model as a reference instead of draft genomes which results in the functional prediction improvement. However, there are the comments that are needed to be addressed in the manuscript before publication.

**Ans: Thank you very much for your views, comments and suggestions.**

1. There are several functional predictors publicly available, the authors should state the reasons for selection of PICRUSt2?

**Ans: In this study, PICRUSt2 was selected since it has a capability to predict approximate functional potential of a community based on marker gene sequencing profiles. PICRUSt2 accurately predicts MetaCyc pathways and phenotypic data as shown in the Douglas et al. [1] and it shows well-prediction performance on HMP data as illustrated in Figure 3. Moreover, PICRUSt2 can also export metabolic functional results as EC numbers and thus can be directly compared with MetGEMs. We clarified this in the revised manuscript (Page 4, Line 80).**

2. Can the authors discuss for further improvement of MetGEMs e.g., can it be applied by Shotgun datasets?
**Ans: Absolutely, MetGEMs toolbox can be applied with Shotgun datasets. We already added the useful information in the Conclusion Section in the revised manuscript (Page 15, Line 291).**

3. The toolbox seems to be limited with the gut microbiome data or not.
**Ans: The MetGEMs toolbox is not limited to the gut microbiome data. It is a versatile toolbox to use with any types of microbiome data. We added the useful discussion in the Conclusion section (Page 15, Line 295).**

4. Can the authors compare the metabolic results between Pan-, Pan-Weight, Core-, Core-Weight? Which one is the suitable for further functional prediction application?
**Ans: Certainly, we already compared the metabolic results between Pan-, Pan-Weight, Core-, and Core-Weight in a form of EC/KO number as seen in Table 2 and their characteristics and performance (Figure 3A). According to the comparative results, here the Core or Core-weight showed higher performance comparing to others (i.e. Pan or Pan-Weight). Moreover, our result shows consistency with Kaehler et al. [2] in which taxonomic weight did not improve a prediction performance indeed. Taken together, Core-Function was therefore selected in our case study for MetGEMs toolbox application for further functional prediction. We added this useful discussion under the Results and Discussion section in the revised manuscript (Page 10, Line 200).**

5. For the better perform the MetGEMs, is possible to include data in the GitHub as well?
**Ans: Thank you very much for your comments and suggestion. We already added the useful data associated with MetGEMs toolbox in GitHub’s repository. Hereby, we already edited this in the revised manuscript (Page 5, Line 92).**

6. Language is needed to be improved, such as “However, there have not yet been used genome-scale models (GEMs) as scaffold toolbox for microbiome analysis at a systematic level.” in the introduction part.

**Ans: Thank you very much for your comments. We already edited these sentences in the Introduction section in the revised manuscript. For English checking, we also revised throughout the manuscript according to your suggestions.**

**References:**

1. Douglas GM, Maffei VJ, Zaneveld JR, Yurgel SN, Brown JR, Taylor CM, et al. PICRUSt2 for prediction of metagenome functions. Nature Biotechnology. 2020:1-5.

2. Kaehler BD, Bokulich NA, McDonald D, Knight R, Caporaso JG, Huttley GA. Species abundance information improves sequence taxonomy classification accuracy. Nature communications. 2019;10(1):1-10.

**Reviewer #2**:

1. In their paper “MetGEMs Toolbox: Metagenome-scale models as integrative toolbox for uncovering metabolic functions and routes of human gut microbiome”, Patumcharoenpol et al. present MetGEMs, a tool for inferring metagenome-wide KEGG Ortholog and Enzyme Commission number abundances by mapping amplicon sequencing data to genome-scale metabolic network reconstructions. They demonstrate an improvement in performance relative to PICRUSt2 based on increased correlation between inferred KO/EC abundances from amplicon sequencing data and paired metagenomics data. While the software appears to be well-written, the methods underlying the software and all of the benchmarking analyses contained in the paper are severely under-described.

**Ans: Thank you very much for your comments. We revised the description of methods underlying the toolbox and added the details of all analysis in the revised manuscript (Pages 15-21).**

2. Increasing the level of detail in the manuscript is very critical to engaging new users of the software, especially because there appears to be no formal documentation for the software in the GitHub repository. Overall, I feel that this is a very valuable contribution, but it will have much higher impact and probability of adoption if the quality and detail of the manuscript is improved.

**Ans: Thank you very much for your comments. We additionally described all possible details and improved quality throughout the revised manuscript. To increase the user’s engagement, we added a formal user guide of MetGEMs toolbox and also in the GitHub’s repository (Page 5, Line 92).**

**Major comments**

3. The authors statements of novelty for their method are overstated and should be made more nuanced. Specifically, the authors seem to be communicating that inference of community-level metabolic functionality from 16S rRNA gene sequencing data has never been performed. For example, in lines 70-72, the authors state the AGORA GEMs “... have not yet been used as a scaffold toolbox to infer metagenomic content from 16S rRNA sequenced samples at a systematic level”, but this was done to a small extent in the original AGORA paper (see the last figure in Magnusdottir et al. 2017 for inference of reaction content from both 16 rRNA gene sequencing data and shotgun metagenomics data). The authors of the AGORA paper have also since performed more in-depth versions of this analysis on many occasions (for example, see Baldini et al., BMC Biology 2020, “Parkinson’s disease-associated alterations of the gut microbiome predict disease-relevant changes in metabolic functions”). The authors’ statements describing the need for their method or the innovative aspects should include this context.

**Ans: We already revised this statement for clarification. We cited previous paper on existing studies that used metabolic models for functional analysis according to your suggestion. In context of our work, we would like to develop a MetGEMs as an open-source integrative toolbox for predicting metabolic functions and routes of human gut microbiome from 16S rRNA gene sequences. We already edited the objective of this work accordingly in the revised manuscript (Page 4, Line 69-75).**

4. Methods are described with insufficient detail for the majority of analyses in the paper. We recommend adding more detail to the description of every analysis (including steps of the MetGEM pipeline as well as all of the analyses used to test MetGEM performance). Here is a non-exhaustive list of examples of issues that I cannot find answers to in the manuscript: 1) In the atopic dermatitis analysis, was Core- or Pan-functionality used to compute KO/ECs?

**Ans: Thank you very much for your comments and suggestion. We already added more details in every step of MetGEMs pipeline as well as all of the analysis steps used to test MetGEMs performance in the Materials and Methods and S1 Doc. The user’s guide of MetGEMs toolbox is already included in the GitHub’s repository (**[**https://github.com/yumyai/MetGEMs/blob/master/README.md**](https://github.com/yumyai/MetGEMs/blob/master/README.md)**). Regarding to atopic dermatitis analysis, we used Core-Function analysis to compute KO IDs and EC numbers. We already added and clarified useful information throughout the revised manuscript (Page 12, Line 236).**

5. No methods are provided for 16S rRNA gene sequence data processing other than the software and databases used. BBDUK, DADA2, and QIIME2 all have many parameters selected by the user, and the authors provide zero parameter values. This needs much more detail to be reproducible. 3) Similar to issue (2), no details for parameters used in HUMAnN2 are provided.

**Ans:** **We apologized for unclear information. We already included the parameters, software, and database versions used for this study throughout Materials and Methods and S1 Doc. We also revised the descriptions MetGEMs pipeline and the analysis steps used to test MetGEMs performance throughout Materials and Methods and S1 Doc to clarify all steps. We already added useful information in the revised manuscript (Page 15-21)**

6. Insufficient detail on bootstrapping is provided. Both the number of samples and the *size* of each sample are needed. Furthermore, in the analysis in Fig S1, I can’t tell what the non-bootstrapped line is supposed to be indicating; the authors should describe this.

**Ans: We apologized for insufficient details. In the revised manuscript, we already provided number of samples and size for each sample for bootstrapping. In addition, we generated a new Fig. S2 for clarification of bootstrapping results**. **We already added useful information in the revised manuscript (Page 11, Line 216-222).**

**Minor comments**

7. Figure 2A-what do the green and blue lines represent? It looks like number of reactions, but it isn’t labelled anywhere. Also, the choice to annotate the figure with the correlation coefficient is very odd--it would be clearer to simply add an additional scatter plot showing the data that went into the correlation, and the goodness of fit for the correlation.

**Ans: The blue line and green line indicate the numbers of KO IDs and EC numbers, respectively. This useful information was added in legend of Figure 2. Regarding on Figure 2A, we already provided additional scatter plot showing the data correlation and the goodness of fit for the correlation between numbers of KO IDs and EC numbers as shown in additional S1 Fig. We also revised the legend of Figure 2 for clarification.**

8. Within the results around lines 127-159, no justification is given for the “weighted” versions of the core/pan metrics, and it’s not clear how they are computed. The use of these metrics should be justified when they are introduced here, and their computation should be briefly described (with more detail in the methods).

**Ans: We apologize for unclear information. We already provided a justification of “weighted” versions (Page 10, Lines 196-200). The explanations and details on the computations of both core/pan metrics and “weighted” version has been improved throughout the revised manuscripts (Page 7, Line 136-139, Pages 15-18).**

9. Figure 1, “Specie” is not the singular form for “species”--both singular and plural forms of the words are “species”. This is unfortunate and confusing, but the authors should correct “Specie” to “Species” to be consistent with English conventions.

**Ans:** **Thank you very much for your suggestion. We already edited the Figure 1.**

10. References to the 16S rRNA gene sequence should use the complete terminology, e.g., “16S rRNA gene sequencing”, and not “16S rRNA sequencing”, since the *gene*

**Ans: Thank you very much for your suggestion. We already edited it throughout the revised manuscript.**

**Have all data underlying the figures and results presented in the manuscript been provided?**
Large-scale datasets should be made available via a public repository as described in the *PLOS Computational Biology* [data availability policy](http://journals.plos.org/ploscompbiol/s/data-availability), and numerical data that underlies graphs or summary statistics should be provided in spreadsheet form as supporting information.

Reviewer #1: None

Reviewer #2: Yes

**Ans:** Thank you very much for comments.

PLOS authors have the option to publish the peer review history of their article ([what does this mean?](https://journals.plos.org/ploscompbiol/s/editorial-and-peer-review-process#loc-peer-review-history)). If published, this will include your full peer review and any attached files.


If you choose “no”, your identity will remain anonymous but your review may still be made public.

**Do you want your identity to be public for this peer review?** For information about this choice, including consent withdrawal, please see our [Privacy Policy](https://www.plos.org/privacy-policy).

Reviewer #1: No

Reviewer #2: No

**Ans:** Thank you very much for comments.

Figure Files:

While revising your submission, please upload your figure files to the Preflight Analysis and Conversion Engine (PACE) digital diagnostic tool, [https://pacev2.apexcovantage.com](https://pacev2.apexcovantage.com/). PACE helps ensure that figures meet PLOS requirements. To use PACE, you must first register as a user. Then, login and navigate to the UPLOAD tab, where you will find detailed instructions on how to use the tool. If you encounter any issues or have any questions when using PACE, please email us at [figures@plos.org](mailto:figures@plos.org).

Data Requirements:

Please note that, as a condition of publication, PLOS' data policy requires that you make available all data used to draw the conclusions outlined in your manuscript. Data must be deposited in an appropriate repository, included within the body of the manuscript, or uploaded as supporting information. This includes all numerical values that were used to generate graphs, histograms etc.. For an example in PLOS Biology see here: http://www.plosbiology.org/article/info%3Adoi%2F10.1371%2Fjournal.pbio.1001908#s5.

Reproducibility:

To enhance the reproducibility of your results, PLOS recommends that you deposit laboratory protocols in protocols.io, where a protocol can be assigned its own identifier (DOI) such that it can be cited independently in the future. For instructions, please see [http://journals.plos.org/compbiol/s/submission-guidelines#loc-materials-and-methods](http://journals.plos.org/plospathogens/s/submission-guidelines)

*In compliance with data protection regulations, you may request that we remove your personal registration details at any time.* [*(Remove my information/details)*](https://www.editorialmanager.com/pcompbiol/login.asp?a=r)*. Please contact the publication office if you have any questions.*
